# Effect of Competitive and Non-competitive Exercise on Serotonin Levels in Adolescents with Various Levels of Internet Gaming Addiction

**Published:** 2018-07

**Authors:** So-Hyung KANG, Wi-Young SO

**Affiliations:** 1. Dept. of Sports and Wellbeing, Arts & Sports, Hanyang University, Ansan-si, Korea; 2. Sports and Health Care Major, College of Humanities and Arts, Korea National University of Transportation, Chungju-si 27469, Korea

## Dear Editor-in-Chief

The excessive progression of internet gaming addiction results in obsessive feelings that worsen as the individual’s tolerance develops and duration of use increases, with adolescents often experiencing withdrawal, among other negative effects (1, 2). Internet gaming addiction may also induce insomnia, eating disorders, decreased ability to exercise and overall fitness, depression, and attention deficit hyperactivity disorder (ADHD) (3). Various medical treatments, as well as art therapy, psychotherapy, and exercise therapy, exert positive effects on clinical symptoms of gaming addiction and ADHD (4, 5).

Although physical activity is known to exert positive effects on gaming addiction, it may be fundamentally difficult for adolescents with sedentary lifestyles to adopt more active patterns of behavior involving vigorous or even simple physical activity, limiting the efficacy of such treatment approaches. Thus, it may be more effective to induce voluntary motivation via application of an exercise program that more accurately reflects the psychological characteristics of adolescents with gaming addiction by developing a competitive environment that simulates a game-like situation.

However, little research has focused on the treatment of internet gaming addiction in adolescents. Investigating the comparative effects of “competitive” and “non-competitive” exercise types in individuals with various levels of gaming addiction may allow for the development of appropriate exercise therapy protocols for this population.

The neurotransmitter serotonin is mainly involved in emotion regulation and the experience of pleasure and is therefore referred to as the “happiness hormone”. Serotonin, in particular, plays important roles in the development of various mental disorders, aggression, suicidal ideation, obsessive-compulsive disorder, appetite, and anxiety (6). Furthermore, serotonin levels seem to be influenced by exercise type as well (6).

In the present study, we aimed to investigate the effect of both competitive and non-competitive exercise on adolescents with various levels of internet gaming addiction. Serotonin levels were measured in order to objectively and quantitatively assess psychological improvement.

Participants were classified into competitive and non-competitive exercise groups. Participants of the competitive exercise group were divided into the following three groups according to scores on the adolescent (13–18 yr old) Game Addiction Scale (7) developed by the Korea National Information Society Agency: addiction group (AG; n=8, age: 17.02±0.20 yr, height: 172.24±3.23 cm, weight: 66.67±10.23 kg), potential addiction group (PAG; n=9, age: 17.00±0.40 yr, height: 171.80±2.35 cm, weight: 65.36±7.46 kg), non-addiction group (NG; n=7, age: 16.95±0.62 yr, height: 174.74±3.65 cm, weight: 67.07±10.35 kg). Participants of the non-competitive exercise group were also divided into an AG (n=7, age: 17.00±0.74 yr, height: 173.76±6.48 cm, weight: 69.28±7.42 kg), a PAG (n=6, age: 17.08±0.68 yr, height: 174.28±3.17 cm, weight: 68.03±9.84kg), and an NG (n=5, age: 17.10±0.25 yr, height: 172.66±4.26 cm, weight: 66.72±8.79 kg).

Informed consent was taken from the participants before the study and local university approved the study ethically.

Adolescents participated in a 12-wk exercise program for three sessions each week at average 70% of maximum heart rate. Table 1 describes the respective protocols for the competitive and non-competitive exercise programs. Blood was drawn (10 mL) from the vein of the forearm prior to and following the 12-week program in order to assess changes in serotonin levels.

**Table 1:** Competitive and Non-competitive exercise programs

***Category***	***Type***	***Methods***
Warm-up (10 min)	Stretching	Upper & lower body stretching
Main exercise (30 min)	Competitive exercise group	Team soccer game Average 70% heart rate max (3 times per week)
	Non-competitive exercise group	Treadmill exercise Average 70% heart rate max (3 times per week)
Cooldown (10 min)	Stretching	Upper & lower body stretching

All results are presented as the mean±standard deviation. Data analyses were performed using 3 × 2 repeated measures analyses of variance. Statistical significance was set at a level of *P*<0.05 and all analyses were performed using SPSS ver. 18.0 (SPSS, Chicago, IL, USA).

Increased serotonin levels were observed following 12 wk of competitive exercise (time, F=40.091, *P*<0.001) as well as 12 wk of non-competitive exercise (time, F=33.572, *P*<0.001) in the AG, PAG, and NG groups. However, no differences were noted among the groups with regard to either competitive (interaction, F=1.803, *P*=0.189) or non-competitive exercise (interaction, F=0.544, *P*=0.592) (Table 2).

**Table 2:** Changes in serotonin concentration after 12 wk of exercise training

***Categories***	***Group***	***Preexecise***	***Postexercise***		***F***	***P***
Competitive exercise	Addiction (n=8)	222.63±113.22	298.98±112.31	Time	40.091	<0.001***
	Potential addiction (n=9)	264.64±56.71	301.12±46.18			
	Non-addiction (n=7)	177.19±71.92	232.75±75.04	Interaction	1.803	0.189
Non-competitive exercise	Addiction (n=7)	170.93±37.18	247.32±50.42	Time	33.572	<0.001***
	Potential addiction (n=6)	217.87±91.29	310.12±65.57			
	Non-addiction (n=5)	191.41±78.45	249.11±66.85	Interaction	0.544	0.592

Data are presented as mean±standard deviation

Serotonin concentration; ng/*ml*

*** *P*<0.001; tested by 3 × 2 repeated measures analysis of variance

Regular exercise may increase blood levels of serotonin and exert positive effects on mood and symptoms of internet gaming addiction, regardless of addiction level.
